# Lernen auf Distanz während der Coronapandemie: Eine Längsschnittstudie zu Ressourcen und Herausforderungen aus Elternsicht

**DOI:** 10.1007/s42278-023-00168-z

**Published:** 2023-02-23

**Authors:** Helen Knauf

**Affiliations:** Fachbereich Sozialwesen, Interaktion 1, Fachhochschule Bielefeld, 33619 Bielefeld, Deutschland

**Keywords:** Grundschule, Distanzunterricht, Corona, Familie, Eltern, Primary school, Distance learning, Corona, Family, Parents

## Abstract

Während der Coronapandemie mussten Eltern intensiver als sonst das Lernen ihrer Kinder begleiten und unterstützen. Zusätzliche Herausforderungen ergaben sich dadurch, dass die Ausnahmesituation nicht nur auf wenige Wochen begrenzt war, sondern sich über mehr als ein Jahr hinzog. Die hier vorgestellte Studie geht der Frage nach, welche Ressourcen und Probleme es aus Sicht von Eltern beim Lernen auf Distanz gab. Dazu wurden Eltern von Grundschülerinnen und -schülern an zwei verschiedenen Zeitpunkten während der Pandemie mit Interviews befragt. Durch das Längsschnitt-Design konnten Veränderungen und Kontinuitäten erfasst werden. Die Untersuchung zeigt, dass für die Bewältigung der Situation sowohl Charakteristika der Familie als auch Merkmale der Schule eine zentrale Rolle spielten. Gerade für Familien mit geringen eigenen Ressourcen spielten unterstützende Schulfaktoren eine zentrale Rolle. Diese lagen vor allem in einer schülerorientierten Unterrichtgestaltung, die die Kinder befähigte, Aufgaben selbstständig zu bearbeiten.

## Abstract (en)

During the Corona pandemic, parents had to accompany and support their children’s learning more intensively than usual. Additional challenges arose from the fact that the exceptional situation was not only limited to a few weeks, but extended over more than a year. The study presented here explores the question of what resources and problems there were in remote schooling from the parents’ point of view. For this purpose, parents of primary school pupils were interviewed at two different points in time during the pandemic. The longitudinal design made it possible to record changes and continuities. The study shows that both characteristics of the family and characteristics of the school played a central role in coping with the situation. Especially for families with few resources of their own, supportive school factors played a central role. These were mainly in the form of student-oriented teaching, which enabled the children to work on tasks independently.

## Schulschließungen als lange Belastung für Schule und Eltern

Schule und Eltern wirken bei der Verwirklichung der Bildungs- und Erziehungsziele partnerschaftlich zusammen (§ 2, Abs. 3 SchulG NRW)

Eine partnerschaftliche Zusammenarbeit zwischen Schule und Eltern soll zum Bildungserfolg der Kinder beitragen. Diese Idee wird an obigem Zitat aus dem nordrhein-westfälischen Schulgesetz sichtbar. Im üblichen Schulbetrieb gibt es deshalb einige neuralgische Punkte, an denen Eltern in den Schulalltag einbezogen werden: Schulpflegschaft, Gestaltung des Schullebens sowie die Sicherstellung des pünktlichen und gut ausgerüsteten Erscheinens der Kinder zum Unterricht. Für die Unterrichtsinhalte ist hingegen in der Regel die Schule zuständig; lediglich bei dem Thema Hausaufgaben gibt es Überschneidungen zwischen den Aufgaben beider Akteure. Aufgrund der wachsenden Bedeutung von Ganztagsangeboten, die typischerweise auch die Hausaufgabenbegleitung beinhalten, nimmt diese Schnittmenge jedoch ab (Knoll 2018).

Einen umso stärkeren Bruch mit der bisherigen Praxis der Elternzusammenarbeit waren deshalb die Phasen der Schulschließung während der Coronapandemie 2020 und 2021. Unterricht in Schulen durfte zeitweise gar nicht oder nur in kleinen Gruppen stattfinden; die Kinder mussten vollkommen oder überwiegend zuhause lernen (im Brahm et al. 2021). Insbesondere für Grundschülerinnen und -schüler war dabei die Unterstützung durch ihre Familien wichtig; Eltern hatten eine neue, wichtige Rolle für die Sicherstellung des Lernens (Porsch und Porsch 2020; Helm et al. 2021; Müller 2020). Dies gelang den Familien unterschiedlich gut (Andresen et al. 2020; Lochner et al. 2021; Porsch et al. 2021; Venard et al. 2022). Befragungen von Kindern zeigen, dass auch sie selbst die Situation unterschiedlich bewerteten (Kirsch und Neumann 2022). Ein Teil der Familien meisterte die Situation gut und konnte ihr auch positive Seiten abgewinnen, wie etwa die gemeinsame Zeit als Familie, der Wegfall von Terminen in Beruf und Freizeit, sowie die intensivere Auseinandersetzung mit den Lerninhalten der Kinder (Langmeyer et al. 2020). Auch das Lernen zuhause hatte aus Sicht eines Teils der Eltern auch Vorteile, weil der Schulbesuch auch mit negativen Erfahrungen verbunden ist: Konflikte mit Lehrkräften und anderen Kindern, Ablenkung durch die Gruppensituation und vor allem die Notwendigkeit, das eigene Lerntempo an die Gruppe anzupassen. Viele Eltern erlebten deshalb ein ausgeprägtes Spannungsverhältnis: Einerseits erfreuten sie sich an dem engeren Kontakt in der Kleinfamilie; andererseits fühlten sie sich ausgelaugt durch die dauerhafte Mehrfachbelastung („Corona-Paradox“, Knauf 2021, S. 14).

Für einen anderen Teil der Familien war die Phase der Schulschließungen und des Wechselunterrichts kaum zu bewältigen (Helm et al. 2021; Huber und Helm 2020; Huebener et al. 2021; Porsch et al. 2021; Wößmann et al. 2021; Zinn und Bayer 2021). Dazu gehörten häufig Familien mit niedrigem sozio-ökonomischem Status, weil sie z. B. nicht über die notwendigen digitalen Endgeräte und ausreichende Netzanbindung verfügten oder die Wohnungen nicht genug Rückzugsmöglichkeiten zum konzentrierten Lernen boten. Besonders anstrengend war die Zeit für Alleinerziehende, die bei Care-Aufgaben weitgehend auf sich gestellt waren. Auch Familien mit Kindern mit Behinderung, die während des normalen Schulbetriebs verschiedene Unterstützung erhalten, waren besonders belastet. Sie mussten mehr Zeit in die Begleitung ihrer Kinder investieren; die Kinder haben nach dem Eindruck ihrer Eltern auch weniger gelernt (Nusser 2021).

Unabhängig vom Erfolg der Bildungsbegleitung empfanden fast alle Eltern die Situation als belastend. Dies lag zum einen daran, dass die Familien aufgrund der Kontaktbeschränkungen den gesamten Tag ausschließlich miteinander verbrachten; die Eltern waren oft allein für Bildung und Betreuung ihrer Kinder zuständig. Zum anderen ergab sich die zentrale Herausforderung dadurch, dass die meisten Eltern ihren Beruf weiterhin voll ausüben mussten (Lochner et al. 2021). Besonders betroffen von dieser Doppelbelastung waren die Mütter (Bujard et al. 2020; Langmeyer et al. 2020). Zwar hatten viele die Möglichkeit, zuhause zu arbeiten und dadurch zumindest räumlich bei ihren Kindern zu sein. Das bedeutete jedoch auch, dass sie Arbeit, Betreuung und Lernbegleitung parallelisieren mussten, was sie stark forderte (Knauf 2020). So ist es wenig überraschend, dass viele Eltern den Eindruck hatten, den Bedürfnissen ihrer Kinder weniger gut gerecht zu werden als vor der Pandemie (Geissler et al. 2022).

Lernerfolg und Wohlbefinden hingen jedoch nicht nur von den familiären Rahmenbedingungen ab. Ein entscheidender Einflussfaktor war die Schule. Die Art und Weise, in der Lehrkräfte den Unterricht gestalteten, erwies sich den aktuell vorliegenden Studien zufolge als ebenso divers wie die häusliche Lernumgebung. Eine Ursache hierfür liegt auch darin, dass die konkrete Umsetzung des Distanzlernens weitgehend den einzelnen Lehrkräften überlassen wurde (Bremm et al. 2021).

Ein Teil der Schulen setzte durchgehend auf die Nutzung von papierbasierten Aufgaben in Form von Arbeitsheften und -blättern (Helm et al. 2021; Kirsch et al. 2021; Porsch und Porsch 2020; Steinmayr et al. 2021; Schütz et al. 2020). Dadurch nahm das Distanzlernen den Charakter umfangreicher Hausaufgaben an und nicht den eines abwechslungsreichen Unterrichts. Andere Schulen bzw. Lehrkräfte setzten selbstgedrehte Videos, Apps, digitale Pinnwände, Videokonferenzen und Lernplattformen ein, wobei im Verlauf der Schließungsphasen der Anteil dieser digitalen Angebote zunahm (Nusser et al. 2021). Dabei war der Prozess der Digitalisierung stark durch die Infrastruktur bestimmt. Im zweiten Lockdown, der Ende 2020 begann, war die Ausstattung von Schulen mit digitalen Endgeräten deutlich weiter fortgeschritten als zu Beginn des ersten Lockdowns. Vielerorts waren beispielsweise in der Zwischenzeit (mehr) digitale Endgeräte in den Schulen vorhanden, Lizenzfragen geklärt und landeseigene Lernplattformen einsatzbereit, wie etwa in Nordrhein-Westfalen die Plattform Logineo.

Insgesamt muss jedoch resümiert werden, dass der Anteil eines Unterrichts, der die Möglichkeiten des Digitalen nutzt, über die gesamte Zeitspanne hinweg klein blieb (Helm et al. 2021). Als Barrieren gegenüber dem Einsatz digitaler Unterrichts- und Kommunikationsformen erwiesen sich die technische Ausstattung der Schulen und mangelnde Erfahrungen von Lehrkräften mit digitalen Angeboten (Anger und Plünnecke 2021). Wie Bossen und Breidenstein (2022) herausarbeiten, war ein wichtiger Grund für diese analoge Orientierung des Unterrichts die Erwartung, mit digitalen Unterrichtsformen nicht alle Schülerinnen und Schüler erreichen zu können. Daneben gab es die Überzeugung, dass die Ko-Präsenz der Lehrkräfte mit den Lernenden eine notwendige Voraussetzung für gelingenden Unterricht ist (ebd.). In Schulen mit einem hohen Anteil von Schülerinnen und Schülern mit niedrigem sozioökonomischem Status kam hinzu, dass Lehrkräfte niedrige Erwartungen an die Nutzung digitaler Unterrichtsformen durch die Lernenden hatten und deshalb auch kaum entsprechende Angebote bereitstellten (Bremm und Racherbäumer 2020).

Der Kontakt zwischen Lehrkräften und Schülerinnen und Schülern war ebenfalls unterschiedlich eng. Insbesondere zu Beginn der coronabedingten Schließungen, also zu einem Zeitpunkt, als noch wenig Erfahrungen mit Distanzunterricht vorlagen und die Dauer der Schließungen ungewiss war, gab es oftmals kaum Kontakt: Über die Hälfte der Schülerinnen und Schüler gab an, nur einmal wöchentlich Kontakt zu ihren Lehrerinnen und Lehrern zu haben (Vuorikari et al. 2020). Zehn Monate später, zu Beginn des Jahres 2021, berichteten weiterhin viele Schülerinnen und Schüler, nie gemeinsamen Unterricht, z. B. als Videokonferenz, zu haben, keine individuellen Gespräche mit ihren Lehrkräften zu führen oder Rückmeldungen auf ihre eingereichten Aufgaben zu erhalten (Wößmann et al. 2021). Insgesamt zeigt sich, dass die Unterschiede zwischen den Schulen sehr groß und die Erfahrungen der Schülerinnen und Schüler dementsprechend vielfältig waren (ebd.).

An der Vielfalt der Erfahrungen setzt die hier vorgelegte Studie an. Ziel war es, die Interaktion zwischen Familie und Schule zu untersuchen, indem insbesondere Veränderungen im Laufe der Pandemiejahre 2020 und 2021 in den Blick genommen werden. Die hier vorgestellte Analyse der Perspektiven und Erfahrungen von Eltern geht den Fragen nach, wie Eltern die Zusammenarbeit mit der Schule wahrnahmen, wie sich dieses Erleben möglicherweise im Pandemieverlauf verändert hat und welche Einflussfaktoren die Wahrnehmung der Eltern prägte.

## Methodisches Vorgehen

Die vorliegende Studie stellt eine Längsschnitterhebung dar. Dafür wurden Interviews mit 16 Müttern von Kindern im Grundschulalter durchgeführt. Die Mütter wurden jeweils zu zwei Zeitpunkten (Mai/Juni 2020 und März/April 2021) während der Pandemie interviewt, so dass insgesamt 32 Interviews in die Analyse einflossen. Beide Befragungszeitpunkte lagen in Anlehnung an Reintjes (2022) in Phasen der „Wiedereröffnung“. Dabei verbrachten die Schülerinnen und Schüler einen Teil der Lernzeit in der Schule und einen anderen Teil zuhause, wobei der Umfang regional und auch in Bezug auf einzelne Schulen stark variierte (ebd.). Dem Wechselunterricht war jeweils eine Phase der kompletten Schulschließung vorausgegangen.

Es wurde ein qualitatives Forschungsdesign gewählt, um Wahrnehmungen und Deutungen von Eltern zu erfassen, ohne dass bereits zuvor festgelegte Kategorien einer standardisierten Erhebung die Antwortmöglichkeiten begrenzen. Der Längsschnitt gerade in qualitativen Studien erlaubt eine besonders differenzierte Analyse, wie sich Wahrnehmungen und Haltungen ändern oder inwiefern und warum sie stabil bleiben (Thiersch 2020).

Die Studie hat die Prinzipien der Grounded Theory Methode (GTM) angewandt; deren Fokus liegt darauf, die Perspektiven der Untersuchungsteilnehmerinnen und -teilnehmer nachzuvollziehen (Breuer et al. 2019). Den Einstieg in die Interviews bot ein offener Erzählimpuls; er diente dazu, die Gesprächspartnerinnen und -partner zu einer längeren Narration anzuregen (Rosenthal und Loch 2002). Um sicherzustellen, dass alle auch aus Forschersicht relevanten Themen angesprochen werden und zur Herstellung von Vergleichbarkeit, wurde ein Gesprächsleitfaden eingesetzt.

Die in der Studie befragten 16 Eltern wurden anhand eines Theoretical Samplings ausgewählt. Das Verfahren folgt dem für die GTM charakteristischen Prinzip des ständigen Vergleichens: Nach den ersten Interviews wurden weitere Beteiligte so ausgewählt, dass sie sich möglichst stark von den ersten unterscheiden, um potenziell kontrastierende Perspektiven zu erfassen (Charmaz und Thornberg 2021). So gelang es, Familien in die Untersuchung einzubeziehen, die sich formal deutlich unterschieden in Hinblick auf Anzahl der Kinder, Zahl der mit den Kindern zusammenlebenden Erwachsenen, Beruf und Bildungsstand der Eltern und Familiensprache. Einschränkend muss gesagt werden, dass ausschließlich Mütter gewonnen werden konnten, die sich als schwerpunktmäßig zuständig für die Begleitung des Distanzlernens verstanden. Dies korrespondiert jedoch mit den Befunden zur geschlechtsspezifischen Arbeitsteilung während der Pandemie (Langmeyer et al. 2020).

Alle 32 Interviews wurden von der Autorin durchgeführt und aufgezeichnet. Wegen der pandemiebedingten Ansteckungsgefahr wurden alle Gespräche am Telefon geführt. Es wird davon ausgegangen, dass sich dieses Setting nicht negativ auf die Aussagekraft der Daten auswirkt (Sturges und Hanrahan 2004). Im Anschluss wurden die Interviews transkribiert und mit Hilfe der Software MAXQDA kodiert. Die Kodierung erfolgte nah am Material; das heißt, dass entsprechend den Prinzipien der GTM die Codes aus dem Datenmaterial heraus entwickelt wurden. Die Codes wurden anschließend zu Kategorien verdichtet. Die im Material identifizierten Kategorien ließen sich in zwei Kategorienbündel gliedern: Ein Kategorienbündel umfasst die Faktoren, die die Familie selbst mitbrachte (Familienfaktoren), ein zweites diejenigen, die durch die Schule an die Familien herangetragen wurden (Schulfaktoren). Familienfaktoren sind die konkreten Zeitressourcen, die Alltagsorganisation, die Zahl der an der Care-Arbeit Beteiligten, die Arbeitssituation, die finanzielle Lage sowie die Wohnsituation; hierzu gehören aber auch das Alter der Kinder, das Wohlbefinden der Kinder, das Belastungsempfinden, das Selbstwirksamkeitsempfinden, die Lebenseinstellung/Weltanschauung, die Einschätzung der Leistungsstärke der Kinder und ihre Motivation zur Bearbeitung der Schulaufgaben, der Umgang mit der neuen Lehrerrolle, familiäre Beziehungen und die konkrete Angst vor Ansteckung mit dem Coronavirus. Schulfaktoren umfassen den Umfang und die Art der Aufgaben, die Einbindung der Eltern in deren Bearbeitung, die Kommunikation mit bzw. durch die Schule, die Klarheit der Aufgaben, die (angemessene) Strukturierung der Aufgaben in Zeiteinheiten, das Eingehen der Lehrkraft auf die individuellen Fähigkeiten des Kindes, die Gestaltung des digitalen Unterrichts, der Anteil (digitalen) Präsenzunterrichts, Rückmeldungen der Lehrkräfte auf die eingereichten Aufgaben, die Lockerheit bzw. Strenge der Lehrkraft und die persönliche Beziehung zur Lehrkraft.

## Ergebnisse

### Verteilung der Befragten im Zeitverlauf

Wie haben die befragten Familien ihre Situation während der Coronapandemie wahrgenommen? Die Kategorienbündel *Familienfaktoren* und *Schulfaktoren* können auf Grundlage der Interviews jeweils als *hoch* oder *niedrig* bewertet werden. Beschrieben die Befragten, dass ihnen für einen Bereich viele oder besonders hilfreiche Ressourcen zur Verfügung standen oder hatten sie den Eindruck, dass sie durch diesen Bereich eher unterstützt und gestärkt wurden, dann wurden sie für dieses Kategorienbündel der Ausprägung *hoch* zugeordnet. Im Gegensatz dazu führte eine Beschreibung eines Kategorienbündels als belastend, Sorgen verursachend und Kräfte zehrend zu einer Zuordnung zur Ausprägung *niedrig*. In analoger Weise erfolgte die Zuordnung zum Kategoreinbündel Schulfaktoren. In der Kombination beider Kategorienbündel und ihrer jeweiligen Ausprägungen ergibt sich die in Tab. 1 dargestellte Matrix. Mit dem Ziel, beide Befragungszeitpunkte miteinander zu vergleichen wurde die Bewertung jeweils auf ein einzelnes Interview bezogen, so dass es möglich war, eine Interviewpartnerin für beide Befragungszeitpunkte unterschiedlichen Feldern in der Matrix zuzuordnen. Auf diese Weise werden Veränderungen und Kontinuitäten sichtbar. Jedes der 32 Interviews konnte einem der vier dargestellten Felder zugeordnet werden.

**Table 1:** 

		Familienfaktoren Wahrnehmung interner Ressourcen	
		Gering	Hoch
Schulfaktoren	Gering	**9, 10, 12, 16** *7, 6, 8, 12, 16*	**1, 2, 3, 4, 5, 6, 7, 8, 11, 13, 14, 15** *1, 4, 13, 14*
Wahrnehmung schulischer Ressourcen	Hoch	*2, 9, 10*	*3, 5, 11, 15*

Die Darstellung in Tab. 1 verdeutlicht, dass schulische Ressourcen während des ersten Lockdowns (Zahlen fett) insgesamt als gering eingeschätzt wurden. Keine der Befragten hatte Schule oder Lehrkräfte als unterstützend oder entlastend empfunden. Unterscheiden konnte man beim ersten Befragungszeitpunkt deshalb nur zwischen Familien mit geringen und hohen eigenen Ressourcen (Familienressourcen). Während 12 der Befragten ihre Ressourcen insgesamt als hoch einschätzten, beschrieben 4 sie als gering.

Am Befragungszeitpunkt 2 (Zahlen kursiv) hat sich die Situation hingegen differenziert. Noch immer waren es 9 Familien, die die Ressourcen der Schulen als gering bewerteten, aber 7 nahmen die Schule als positive Ressource wahr.

Schaut man auf die Veränderungen, dann blieben 6 Befragte in dem Feld, dem sie schon zum ersten Befragungszeitpunkt zugeordnet wurden, während 10 Befragte eine Veränderung durchliefen.

Durch die Längsschnitt-Untersuchung lassen sich insgesamt 6 verschiedene Verläufe identifizieren. Zwei Verläufe waren dabei über die gesamte Zeit persistent:

- Dauerhaft ressourcenarm: Geringe Familien- und Schulfaktoren (*n* = 2) (Abschn. 3.2.1)
- Resilient: Hohe Familienfaktoren, geringe Schulfaktoren (*n* = 4) (Abschn. 3.2.2)

In den anderen vier Verläufe zeigt sich eine Entwicklung:

- Späte Hilfe: Dauerhaft geringe Familienfaktoren konnten im Laufe der Zeit durch hohe Schulfaktoren ausgeglichen werden (*n* = 2) (Abschn. 3.2.3)
- Erschöpfung: Zunächst hohe Familienfaktoren waren zunehmend erschöpft und trafen auf kontinuierlich geringe Schulfaktoren (*n* = 3) (Abschn. 3.2.4)
- Kompensation: Zunächst hohe Familienfaktoren waren zunehmend erschöpft, konnten durch hohe Schulfaktoren ausgeglichen werden (*n* = 1) (Abschn. 3.2.5)
- Verbesserung: Hohe Familienfaktoren wurden zum zweiten Befragungszeitpunkt durch hohe Schulfaktoren ergänzt (*n* = 4) (Abschn. 3.2.6)

Denkbar wäre es auch gewesen, dass es eine Veränderung von geringen Familienfaktoren zu hohen Faktoren gab (z. B., indem ein neuer, tatkräftiger Partner in die Familie zieht oder eine Familie plötzlich zu Reichtum kommt). Unter den hier einbezogenen Familien trat diese Entwicklung nicht ein.

### Veränderungen im Pandemie-Verlauf

#### Dauerhaft ressourcenarm: Geringe Familien- und Schulfaktoren

Zwei der befragten Familien konnten über beide Befragungszeiträume hinweg nur auf wenige Ressourcen zurückgreifen. Bereits im ersten Lockdown fühlten sie sich stark gestresst und kaum in der Lage, ihre Kinder auf angemessene Weise zu begleiten. In beiden Fällen waren die Mütter ganz oder weitgehend allein für die Kinder zuständig. Eine der Mütter war geflüchtet und befand sich selbst noch dabei, sich in die deutsche Sprache und Gesellschaft hineinzufinden. Die andere Mutter war berufstätig und musste Kinderbetreuung und Schulbegleitung allein parallel zum Homeoffice organisieren. Schon während der ersten Schließungsphase war sie dazu übergegangen, abends und am Wochenende zu arbeiten, wenn ihr Mann die Kinder betreuen konnte. Von beiden Müttern wurden die Kinder als motiviert beschrieben, zugleich aber als noch nicht eigenständig und konzentriert genug, um die von der Schule gestellten Aufgaben ohne Anleitung zu verstehen und zu erledigen. Die herausfordernde und ressourcenarme familiäre Situation veränderte sich bis zum zweiten Untersuchungszeitpunkt nicht. Vielmehr nahm die Erschöpfung durch die dauerhafte bzw. wiederkehrende Belastung zu. Insgesamt waren die Familien in dieser Gruppe nach dem zweiten Lockdown sehr erschöpft, wie dieses Zitat verdeutlicht:

Wir sind wirklich alle an dem Punkt, dass wir einfach nur noch erschöpft sind und irgendwie auch so dieser ganzen Beschränkung, so sehr wir sie nachvollziehen können und so sehr die Kinder das mittlerweile auch verinnerlicht haben, aber wir sind dem einfach müde. (t2_16_1)

Die ungünstigen Familienfaktoren trafen in beiden Fällen auf geringe schulische Ressourcen. Der Unterricht bestand ausschließlich darin, dass die Lehrkräfte Aufgaben an die Familien gegeben haben. Dieser Modus veränderte sich bis zum zweiten Erhebungszeitraum nicht. Die Kinder konnten in diesen Familien die Aufgaben nicht allein lösen, sie waren auf die Begleitung durch einen Erwachsenen angewiesen. Die Mütter konnten dies nicht leisten, entweder weil sie keine Zeit dafür hatten oder weil sie sich inhaltlich nicht dazu in der Lage sahen. Die Schule wurde nicht als Hilfe, sondern als zusätzliche Belastung wahrgenommen. Diese Situation änderte sich auch im zweiten Lockdown nicht: Die Schule stellte keine zusätzlichen oder neuen Ressourcen bereit.

#### Resilient: Hohe Familienfaktoren, geringe Schulfaktoren

Ein weiterer Teil der Befragten erlebte über beide Schulschließungsphasen hinweg Kontinuität. Auch ihre Beschreibungen lassen auf geringe Ressourcen durch die Schule schließen; die familiären Ressourcen waren jedoch dauerhaft auf hohem Niveau. In allen vier Familien dieser Gruppe konnten die Mütter die Begleitung des Schulunterrichts so organisieren, dass sie mit ihrer Berufstätigkeit vereinbar war (vor allem durch hohe Zeitautonomie). Dies war möglich, weil die Familien keine existenziellen Sorgen hatten und das Familieneinkommen weitgehend gesichert war. Zudem lebten in allen Familien beide Elternteile in der Familie und Aufgaben konnten auch untereinander aufgeteilt werden. Alle Kinder hatten Geschwister und die Beziehungen unter den Kindern wurden als Ressource beschrieben. So machten sich die Eltern auch keine Sorgen um mangelnde Peer-Beziehung der Kinder.

Bedeutsam ist die positive Entwicklung der Kinder bei der Bearbeitung der schulischen Aufgaben. Die Kinder wurden von ihren Müttern entweder grundsätzlich als erfolgreiche Schülerinnen und Schüler beschrieben oder haben von der häuslichen Lernsituation so profitiert, dass sie sich dazu entwickelt haben, wie in diesem Beispiel:

Also meine Kinder haben beide sehr große Fortschritte gemacht. Gerade beim Kleinen war es vor dem ersten Lockdown auch sehr heikel noch schulisch und da hat er große Schwierigkeiten gehabt. Der Knoten platzte dann so im ersten Lockdown. Und seitdem blüht er schulisch auf. Also auch dann im Präsenzunterricht. Und gehört jetzt tatsächlich zu den Besten. (t2_14_16)

Die schulischen Ressourcen hingegen haben sich im Laufe der Zeit kaum verändert. Es kamen zwar vereinzelt Videokonferenzen als Unterrichtsform hinzu, insgesamt blieb aber das Verteilen von Aufgabenpaketen die typische Form des Unterrichts. Unterschiede gab es dabei in der Strenge, mit der die Schule die Einreichung von Aufgaben einforderte. Dabei betonten die Eltern, bei denen wenig eingefordert wird, dass sie dies als entlastend empfanden, während die Eltern mit sehr strengen Fristen unter dem dadurch entstandenen Druck litten. Insgesamt blieb die Kommunikation aber meist auf E‑Mails zwischen Eltern und Lehrkräften beschränkt.

#### Späte Hilfe: Durch hohe Schulfaktoren konnten geringe Familienfaktoren ausgeglichen werden

Zwei der Befragten, die durchgehend über geringe Familienressourcen berichteten, konnten im zweiten Lockdown auf verbesserte schulische Ressourcen zurückgreifen. Zum ersten Befragungszeitraum beschrieben diese Familien ihre Situation als belastet. Sie fühlten sich wenig kompetent bei der Förderung ihrer Kinder und waren mit der Organisation des Alltags sowie der Kombination mit ihrer Berufstätigkeit überfordert. Eine der Befragten musste zudem allein für ihr Kind sorgen und war zudem noch für ihren hochaltrigen Vater verantwortlich. Beide Mütter beschrieben schon am ersten Befragungszeitpunkt, dass es schwer war, die Kinder zum Arbeiten für die Schule zu bewegen. Auch hatten die Befragten den Eindruck, in einzelnen Fächern den Kindern nicht optimal helfen zu können. In einem Fall war die Mutter auch von der Notwendigkeit der Corona-Schutzmaßnahmen nicht überzeugt, was bei ihr zusätzlichen Stress auslöste.

Bei der zweiten Befragung hatte sich die Schule als in mehrfacher Hinsicht hilfreich erwiesen. Zwar berichteten beide Mütter, dass weiterhin kein Online-Unterricht stattfand; es wurden aber zusätzliche digitale Werkzeuge eingesetzt, wie etwa eine Kommunikations-App, die eine niedrigschwellige Kontaktaufnahme zur Lehrkraft ermöglichte. Dieser Kontakt wurde als besonders hilfreich empfunden:

Schon allein mit den Gesprächen mit der Klassenlehrerin. Also, einmal in der Woche haben wir uns mal gesehen, Aufgaben abgegeben und neue abgeholt. Und da hat sie sich wirklich Zeit genommen und alles schön in Ruhe erklärt und alle Fragen beantwortet. Und nach meiner Meinung war das unterstützend, vor allen Dingen hat sie selbst mir die Notbetreuung angeboten. (t2_10_38)

Das Beispiel zeigt auch, dass die Schule durch das offensive Anbieten von konkreter Unterstützung (hier: Notbetreuung) eine Hilfe war. Eine stärkere Strukturierung der Aufgaben für die Kinder im zweiten Lockdown wurde als deutliche Verbesserung empfunden:

Von der Schule her wurde vieles besser organisiert. Also, dass man eben regelmäßig die Lernpakete abholt, sodass die Lehrer dann zum Beispiel … Also zumindest für Deutsch und Mathe waren dann konkrete Stundenpläne für jeden Tag aufgeschrieben, also an welchem Tag die Kinder was machen sollten, für welches Fach, und das hat unserer Tochter sehr, sehr geholfen, diese Struktur, die da drin war. (t2_9_11)

Gerade in diesen beiden Familien, die sich in der Phase der Schulschließungen belastet fühlten und ihre Kinder selbst nur unzureichend unterstützen konnten, erwies sich die Schule während des zweiten Lockdowns mit relativ einfachen Mitteln als eine hilfreiche Ressource.

#### Erschöpfung: Zunächst hohe Familienfaktoren sind zunehmend erschöpft und treffen auf kontinuierlich geringe Schulfaktoren

Einige Familien, die während der ersten Schulschließungsphase noch auf ausgeprägte eigene Ressourcen zurückgreifen konnten, befanden sich bei der zweiten Befragung in einer problematischen Situation, denn ihre Reserven waren erschöpft. Die Befragten dieser Gruppe konnten der Schließung im ersten Lockdown noch positive Seiten abgewinnen. Ihnen gelang es, die Anforderungen miteinander zu verbinden und so in der Summe eine positive Erfahrung zu gestalten. In je unterschiedlichen Konstellationen konnten die Eltern sich so organisieren, dass sie Zeit für ihre Kinder hatten. Im zweiten Lockdown war dies für die Befragten aus verschiedenen Gründen nicht mehr möglich, so dass sich die familiären Ressourcen in diesen Familien im Laufe der Zeit verringerten. Auch das lange Andauern von Distanz- und Wechselunterricht reduzierte die Energie. Diese Verschlechterung konnte durch die Schule nicht ausgeglichen werden, sondern wurde durch diese zum Teil sogar noch verstärkt. Aufgabenumfang und -anforderungen wurden von den Befragten als sehr hoch beschrieben, wie dieses Beispiel zeigt:

Ich fand es nur ziemlich viel, was die auf hatten. Ich glaube, für meinen Sohn war es sehr anstrengend und für mich auch. Also ich war da manchmal echt überfordert in der Situation. (t2_8_10)

In der Kombination von umfangreichen Aufgaben und weitgehender Überantwortung der Lernbegleitung an die Eltern entstand so eine Belastung, die die Eltern nicht mehr auffangen konnten.

#### Kompensation: Zunächst hohe Familienfaktoren sind zunehmend erschöpft, können durch hohe Schulfaktoren ausgeglichen werden

Ein anderer Entwicklungsverlauf ist dadurch charakterisiert, dass die zunächst großen Familienressourcen zunehmend erschöpft waren, die Schule sich aber als hilfreich erwies und deshalb ausgleichend wirken konnte. Unter den im Rahmen dieser Studie Befragten wies nur eine Familie diesen Verlauf auf. Eine wesentliche Veränderung ergab sich hier durch die Geburt eines weiteren Kindes zwischen den beiden Lockdowns. Die für die Kinderbetreuung schwerpunktmäßig verantwortliche Mutter musste deshalb die Versorgung eines Babys und ihrer anderen Kinder mit ihrer eigenen Berufstätigkeit in Einklang bringen, was im Laufe der Zeit immer anstrengender wurde, wie die Mutter zusammenfasst:

Ja, also, die letzten Monate seit dem Lockdown, seit Dezember, muss ich sagen, sind wir schon echt über dem Limit. Also, unsere Energie ist verbraucht. Es war wirklich super, super anstrengend die letzten Monate mit dem Homeschooling. Die Kombination macht es halt bei uns … Ja, wir haben nun mal vier Kinder, ein kleines Baby, Homeschooling und dann auch noch zwei andere, die beschäftigt werden wollen. (t2_2_1)

Angesichts dieser herausfordernden Gesamtsituation waren die Ressourcen, die die Schule bereitstellte, besonders willkommen. Es waren insbesondere drei Elemente, durch die sich die Befragte besonders unterstützt fühlte: Erstens strukturierte die Lehrerin den Tag mit einer allmorgendlichen Videokonferenz, bei der innerhalb von 30 min die Aufgaben des Tages erklärt wurden und der Kontakt zwischen den Kindern gehalten wurde. Zweitens gab es über die eingesetzte Lernplattform eine niedrigschwellige Kontaktmöglichkeit (etwa für Fragen zu den Unterrichtsinhalten) mit der Lehrerin. Zugleich konnten die Kinder die Lernplattform aber auch für die Zusammenarbeit untereinander nutzen. In diesem Fall haben sich kleine Lerngruppen gebildet, bei denen die Kinder miteinander arbeiten konnten und zugleich ihre Beziehung pflegen konnten. Und drittens reagierten die Lehrkräfte bei Problemen zügig und auf nützliche Weise.

Auf diese Weise gelang es der Schule, die Eltern zwar nicht komplett von der Lernbegleitung zu befreien, aber durch Struktur, Erreichbarkeit und Responsivität eine gute Unterstützung zu ermöglichen.

#### Verbesserung: Hohe Familienfaktoren werden zum zweiten Befragungszeitpunkt durch hohe Schulfaktoren ergänzt

Bei diesem Typus wurden die gut ausgeprägten Ressourcen der Familien im zweiten Lockdown durch zusätzliche schulische Ressourcen ergänzt. Die Familien in dieser Gruppe hatten verschiedene potenzielle Belastungsfaktoren zu bewältigen: So gab es zwei Alleinerziehende, in einer anderen Familie waren beide Eltern in Vollzeit erwerbstätig. Zugleich gab es in diesen Familien auch zahlreiche Ressourcen. So beschrieben alle ihre Kinder als leistungsstark, teilweise auch als selbstständig und motiviert bei der Aufgabenbearbeitung. In zwei Familien konnten zusätzliche Personen (Großmutter, befreundete Familie) in die Sorgearbeit verlässlich einbezogen werden. Diese Ressourcen blieben auch im zweiten Lockdown bestehen. Teilweise wunderten sich die Befragten selbst, wie gut sie mit der Situation umgehen konnten. Bei genauerem Hinsehen wird deutlich, dass diejenigen, die diesen Entwicklungsverlauf aufwiesen, ihren Alltag besonders gut organisiert hatten und offenbar auch in der Lage waren, die herausfordernde Situation anzunehmen und ihre Abläufe anzupassen. Dadurch entwickelten sie neue Routinen, die auch bewirkten, dass ihre Kinder das Absolvieren der Schulaufgaben nicht in Frage stellten und nicht immer wieder neu darum gerungen werden musste.

Die Schule, auf der sie ja ist, ist natürlich eine super tolle Schule. Es war wirklich sehr strukturiert und auch sehr organisiert gewesen, im Gegensatz zu anderen Schulen, muss man wirklich sagen. Auch Hut ab an die Lehrer, die wirklich immer mit Leib und Seele, auch im Online-Unterricht dabei gewesen sind. Das war wirklich sehr schön (t2_11_5)

Dieses Zitat verdeutlicht, dass Organisation, ausgeprägte Struktur des Unterrichts sowie das Engagement der Lehrkräfte positiv wahrgenommen wurden. Aber auch die Inhalte des Unterrichts bewerteten die Eltern positiv.

## Diskussion und Fazit

Die befragten Familien kamen während der Phasen von Schulschließung und Wechselunterricht unterschiedlich gut mit den damit verbundenen Belastungen zurecht. Mit diesem Ergebnis schließt die vorliegende Studie an verschiedene andere Untersuchungen an (Andresen et al. 2021; Helm et al. 2021; Porsch et al. 2021; Wößmann et al. 2021; Vuorikari et al. 2020). Deutlich wird durch die längsschnittliche Anlage, dass dies insbesondere auch über die lange Dauer der Pandemiesituation zutrifft: Einzelne Familien konnten in unterschiedlichen Abschnitten der Pandemie die Herausforderungen unterschiedlich gut meisten. Insofern spiegelt die Heterogenität der Erfahrungen und des Erlebens in der Situation die Vielfalt familiärer Lebenslagen insgesamt wider.

Deutlich wird auch, dass ein Teil der Familien die Situation trotz aller Belastungen bewältigt hat und sogar positive Effekte beobachten konnte. Dies traf insbesondere auf jene Familien zu, die bereits mit guten Voraussetzungen in die Krise hineingegangen sind, die also über hohe Familienfaktoren verfügten. Diese Familien waren relativ unabhängig von dem, was die Schule bot bzw. forderte. Ein anderer Teil der Familien war jedoch dringend auf die Unterstützung durch die Schule angewiesen. Für Eltern und Kinder, die bereits vor Pandemiebeginn nur über geringe Familienfaktoren verfügten oder im Laufe der Krise immer weniger auf eigene Ressourcen zurückgreifen konnten, kann die Schule eine hilfreiche Funktion einnehmen und ausgleichend wirken. Diese Familien waren sehr auf das unterstützende Verhalten der Schule angewiesen.

Die vorliegende Studie unterliegt verschiedenen Limitationen. Wesentlich ist dabei die für qualitative Studien charakteristische kleine Stichprobe, die keine Allgemeingültigkeit beanspruchen kann. Dies bedingt auch möglich Verzerrungen, da nur eine begrenzte Vielfalt von Lebenssituationen, Familienformen und auch Schulerfahrungen einbezogen werden konnte. Dieser Begrenzung wird begegnet, indem verschiedenen Typen der Wahrnehmung herausgearbeitet werden, aber keine quantitative Gewichtung oder verallgemeinerbare Zusammenhänge etwa mit sozioökonomischen Parametern hergestellt werden. Dieser Schritt könnte in anknüpfenden Untersuchungen vollzogen werden.

Die in dieser Studie untersuchten Familienfaktoren sind durch Schule nicht oder nur schwer veränderbar: Materielle Armut kann zwar durch die Bereitstellung eines digitalen Endgerätes gelindert werden, die Wohnsituation oder prekäre Arbeitsverhältnisse hingegen entziehen sich dem Einfluss von Lehrkräften. Wirksam sein kann Schule aber dort, wo schulisches Lernen im Vordergrund steht, und zwar in Form der folgenden relevanten Faktoren:

- Aufgaben, die an die individuelle Leistungsfähigkeit des Kindes angepasst sind, so dass die Kinder diese selbstständig bewältigen können.
- Strukturierung des Unterrichtsstoffes in Zeiteinheiten, so dass Kinder sich nicht über- oder unterfordern.
- Differenzierte und zeitnahe Rückmeldungen auf eingereichte, asynchron erledigte Aufgaben.
- Synchroner Online-Unterricht, der die Kinder für eine gewisse Zeit verbindlich beschäftigt und zugleich soziale Kontakte ermöglicht.
- Ansprechbarkeit und Erreichbarkeit durch niedrigschwellige Kontaktmöglichkeiten, die nicht darin besteht, dass eine Telefonnummer oder E‑Mail-Adresse angegeben ist.
- Ermutigung und Gelassenheit bei der Einforderung von Arbeitsergebnissen sowie Verständnis für Verzögerungen oder alternative Bearbeitungsweisen.

Die Aufzählung verdeutlicht, dass es insbesondere um Maßnahmen geht, die sehr nah am Unterrichtsgeschehen sind und weniger um Zusatzangebote oder beziehungsstärkende Aktivitäten. Diese Maßnahmen sind für *alle* Familien hilfreich und nicht nur für jene Familien mit geringen Familienfaktoren. Über die Ausnahmesituation der Coronapandemie hinaus erweisen sich die von den Eltern kritisierten bzw. positiv hervorgehobenen Aktivitäten der Schule im Distanzunterricht als Faktoren, die auch im Normalbetrieb bedeutsam sind. Sie schließen an schulpädagogische Leitvorstellungen von gutem Unterricht (Meyer 2016) an.

Die im vorliegenden Beitrag herausgearbeiteten Typen zeigen zugleich, dass es vor allem auch individueller Angebote bedarf, z. B. der Möglichkeit, sich ein Thema im persönlichen Gespräch noch einmal erklären zu lassen. Die Typologie verdeutlicht, dass nicht alle Familien dieselben (Unterstützungs‑) Angebote benötigen.
